# Perturbation of cellular immune functions in cigarette smokers and protection by palm oil vitamin E supplementation

**DOI:** 10.1186/1475-2891-12-2

**Published:** 2013-01-03

**Authors:** Zakiah Jubri, Azian Abdul Latif, Abdul Gapor Md Top, Wan Zurinah Wan Ngah

**Affiliations:** 1 Department of Biochemistry, Faculty of Medicine, The National University of Malaysia, Kuala Lumpur, Malaysia; 2 Department of Anatomy, Faculty of Medicine, The National University of Malaysia, Kuala Lumpur, Malaysia; 3 Malaysian Palm Oil Board, Bangi, Selangor, Malaysia; 4 Department of Biochemistry, Faculty of Medicine, University Kebangsaan Malaysia, Jalan Raja Muda Abdul Aziz, 50300, Kuala Lumpur, Wilayah Persekutuan, Malaysia

**Keywords:** Tocopherol, Tocotrienol, Cellular immune functions, Cigarette smoke

## Abstract

**Background:**

Cigarette smoke contains free radicals and an have adverse effect to the immune system. Supplementation of palm oil vitamin E (palmvitee), is known has antioxidant properties is thought to be beneficial for system immune protection against free radicals activity. The objective of the study was to determine the effect of palmvitee supplementation on immune response in smokers.

**Methods:**

This study involved a group of smokers and nonsmokers who received 200 mg/day palmvitee and placebo for the control group. Blood samples were taken at 0, 12 and 24 weeks of supplementation. Plasma tocopherol and tocotrienol were determined by HPLC, lymphocyte proliferation by lymphocyte transformation test (LTT) and enumeration of lymphocytes T and B cells by flow cytometry. Statistical analysis was performed by Mann–Whitney *U*-test for non-parametric data distribution and correlation among the variables was examined by Spearman.

**Results:**

Plasma tocopherol and tocotrienol were increased in vitamin E supplemented group as compared to placebo group. Urine cotinine levels and serum α_1_-antitrypsin were significantly higher in smokers compared to nonsmokers. Lymphocyte proliferation induced by PHA showed an increasing trend with palmvitee supplementation in both smokers and nonsmokers. Natural killer cells were decreased; CD4^+^ cells and B cells were increased in smokers compared to nonsmokers but were unaffected with vitamin E supplementation except in the percentage of B cells which were increased in nonsmokers supplemented palmvitee compared to placebo. CD4^+^/CD8^+^ ratio was increased in smokers compared to nonsmokers. The high TWBC count observed in smokers correlated with the increased CD4^+^ and B cells.

**Conclusions:**

Smoking caused alterations in certain immune parameters and palmvitee supplementation tended to cause an increase in lymphocytes transformation test but had no effect on CD3^+^, CD4^+^, CD8^+^, NK cells and B cells except B cells percentage in nonsmokers.

## Background

Studies have reported that cigarette smoking cause impairment of the immune function resulting in diseases such as chronic obstructive lung disease, cardiovascular disease and cancers [1,2]. Cigarette smoke alter immunological functions that affect both humoral and cell-mediated immune responses [3] such as elevated white blood cell count, increased numbers of circulating lymphocytes [4] and an abnormal T-cell profile [5]. Invariant natural killer (iNKT) that regulate and initiate antitumor responses is reported reduced in smokers [3,6]. Proteomics and transcriptomic studies also reveal that protein and genes involves in immune function were altered by smoking [7,8]. The alteration of immune function in term of numbers and proportions of T-cell subsets in the blood of smokers also depending on the amount of cigarette smoking [5,9]. About 10^15^ free radicals in the gas phase of each inhalation from cigarette smoke [10] contribute in initiating and enhancing lipid peroxidation [11], inducing single strand breaks in DNA, oxidizing pulmonary proteins such as α_1_-proteinase inhibitor [12] and also, nicotine in cigarette smoke may induce immunosuppression [13]. Studies on proliferative response of human lymphocytes to phytohemagglutinin (PHA) and LPS was reported decreased [14] and increased [3] in cigarette smoking. Further studied by McCue *et al.*[15] reported that hydroquinone and catechol in cigarette smoke inhibit ribonucleotide reductase and reduces T cell ability to proliferate and lead to cell cycle arrest. B lymphocyte proliferation induced by LPS was also inhibited by cigarette smoke exposure in mice might be cause by the increased superoxide and hydrogen peroxide generation of cigarette smoke [16]. Although many studies reported the harmful effect of smoking, mechanism to overcome this problem is still not yet confirm.

Palm oil vitamin E (palmvitee) is a natural vitamin E consists a mixture of 40% tocopherols and 60% tocotrienols of which γ-tocotrienol is the major component. Each of them consisting of four different forms: α-, β-, γ- and δ-. Study claimed that tocotrienol give better antioxidant activity [17] and immunomodulatory activity on T cell proliferation and cytokine production [18] than tocopherol but tocopherol is the most abundant in nature. Study also has demonstrated that supplementation with alpha-tocopherol alone increased the proliferation of lymphocytes in the presence of LPS [19] and the combination from both might give better effect for disease prevention, treatment or specifically might increase immune response for smokers to reduce the risk from obstructive lung disease, cardiovascular disease and cancers.

Mechanism of vitamin E to increase immune response is still not clearly understood. In healthy elderly persons, the immunostimulatory effect of vitamin E may be mediated due to the ability of vitamin E to decrease prostaglandin (PGE_2_) production [20] and/or decrease other lipid peroxidation products. In smokers isoprostanes which are stable products of lipid peroxidation have been reported to increase [21] suggesting that smokers may benefit from vitamin E supplementation. The aim of this study is to determine the effect of palmvitee supplementation on the immune status of smokers and nonsmokers by measuring the parameters of cell mediated immunity including lymphocytic activity.

## Methods

### Subjects

114 healthy males volunteers aged between 20-50 years were recruited for the study. Each volunteer was briefed on the objectives, design and protocol of the study before informed consent was obtained. The experiments were approved by the Ethics Committee, Faculty of Medicine, UKM. All subjects were healthy, not on any form of treatment and not taking any vitamin supplements through the administration of a questionnaire and dietary interview. The smokers group consisted of individuals who have smoked for 5 years or more and smoking 10 cigarettes or more per day.

### Study design

A randomized single blind placebo controlled study was designed consisting 58 smokers and 56 nonsmokers. The 58 smokers were divided into 2 groups where 28 received placebo while the other 30 received 200 mg/day palmvitee (Palm Oil Research Institute of Malaysia, PORIM). Each capsule of palmvitee contained 60% α-, γ-, and δ-tocotrienols and 40% α-tocopherol. The fifty-six nonsmokers were also divided into 2 groups of which 27 received placebo and another 29 received palmvitee capsules. Blood was withdrawn at 0 week (before the start of supplementation), 12 and 24 weeks of supplementation. Urine was collected in the morning at 0 week and 24 weeks for cotinine measurement. Urine samples were collected into universal bottles containing a few crystals of thymol as preservative. All the urine samples were stored at –20°C. Blood (10 mls) was collected for lymphocyte transformation test (LTT) and T cell profile.

### Plasma tocopherol and tocotrienol determination

Plasma tocopherol and tocotrienol were determined by the method of Meydani *et al.*[22]. Plasma samples were deproteinized with ethanol containing 0.01% BHT. After centrifugation, the supernatant was extracted with 5 volumes hexane (HPLC grade) (Merck, Germany). The hexane layer was removed, dried and redissolved in ethanol. HPLC separations were performed on a silica column (250 × 4.6 mm) preceded by a guard silica column (30 × 4.6 mm) (Supelco, USA) with a mobile phase of hexane:isopropanol (99:1) at a flow rate 1.5 ml/min.

### Cotinine to creatinine ratio (CCR) determination

Urinary cotinine concentrations were measured using the method of Peach *et al.*[23]. The optical densities of the specimens tested by the barbituric acid method were measured at 506 nm using UV-160A visible spectrophotometer (Shimadzu, Japan) 20 min after reaction and compared with the reading given by an aqueous solution of 10 μg/ml cotinine standard. The results were expressed as a ratio of cotinine to creatinine (CCR) μg/mg to compensate for the effect of diuresis. Creatinine was determined by the Jaff reaction [24].

### Serum α_1_-antitrypsin determination

α_1_-Antitrypsin in serum was determined using the method published by Behring Diagnostic, Germany. N Protein standard SY (human), N/T protein contol SY/M (human), N antiserum to human α_1_-antitrypsin and Behring Nephlometer 100 Analyzer were obtained from Behring Diagnostic Germany.

### Lymphocyte transformation test (LTT)

Peripheral blood mononuclear cells (PBMN) were obtained from diluted defibrinated blood by two-fold separation over ficoll hypaque (Pharmacia, USA). The recovered cells were washed, counted and adjusted to a concentration of 2 × 10^6^ cell/ml in RPMI-1640 (Flow Labs, Sydney, Australia); supplemented with 15% heat-inactivated AB serum and 20 ml kanamycin. Con A and PHA (Sigma, St. Louis, USA) inducer cell T activity in 1:5, 1:10, 1:100 and 1:10, 1:50, 1:100, 1:200, 1:400 dilution respectively were set up in culture medium supplemented with 15% AB serum. PBMN were plated in 96-well disposable plates (Nunclon, Denmark) in serial dilution with/without Con A and PHA. PBMN were incubated for 72 hrs at 37°C in 5% CO_2_ incubator. Tritiated thymidine was added 4 hrs before harvesting and the cells counted using a β counter (Wallac, Finland). Dilution Con A 1:5 and dilution PHA 1:50 was chosen for the analysis. Results were obtained in counts per min (CPM) and changed to S.I. (Stimulation Index) = CPM with mitogen/CPM without mitogen.

### T and B cell enumeration

Total white blood cells, total number and the percentage of lymphocyte in whole body were determined using coulter counter T540. For determination of CD3^+^ cells (whole T cells), CD19^+^ cells (B lymphocytes), CD16^+^ and CD56^+^ cells (natural killer cells), CD4^+^ cells (T-helper cells), CD8^+^cells (T-suppressor cells), cells were reacted with monoclonal antibodies. All the monoclonal antibodies were purchased from Becton Dickinson, (USA). The method used involved lysing the erythrocytes with 10% lysing solution followed by washing with PBS. The mixture was vortexed, centrifuged and the supernatant removed. The cells were kept in 1% formaldehyde in PBS and counted using a flow cytometer (Becton Dickinson) and expressed as a percentage of the total white blood cells. Becton Dickinson Simultest™ IMK-*Lymphocyte* is a two-colour direct immunofluorescence reagent kit for enumerating percentages of the mature human leucocyte subsets in erythrocyte-lysed whole blood.

### Statistical analysis

All results are expressed as the mean ± SD. Statistical significance was calculated by using Mann–Whitney *U*-test for nonparametric data distribution. Correlation among the variables was examined by using Spearman. Data analysis was performed using SPSS for Windows, version 17.

## Results

### Plasma total vitamin E level

**Figure 1:**
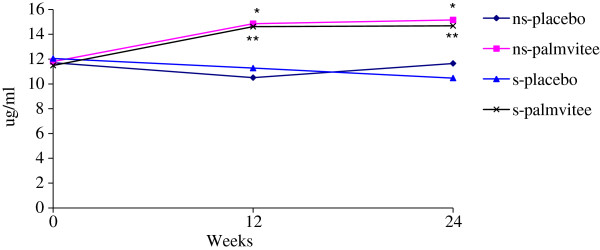
Total plasma vitamin E concentration in smokers and nonsmokers.

There was no difference in total plasma vitamin E, tocopherol and tocotrienol levels in smokers and nonsmokers at the beginning of the study (Figure 1 & Table 1). After palmvitee supplementation, both plasma tocopherol and tocotrienol concentration were increased in smokers and nonsmokers (p < 0.05) starting from 12 weeks until the end of the experiment. Tocopherol concentration was higher in plasma as compared to tocotrienol. Urinary cotinine and serum α_1_-antitrypsin of smokers were significantly higher (p < 0.05) compared to nonsmokers (Table 1) but no changes were observed with supplementation. Urinary cotinine levels were increased accordingly to the number of cigarettes per day (Table 2).

**Table 1:** **The effects of palmvitee supplementation on plasma tocopherol and tocotrienol,** α_**1**_**-antitrypsin in serum and urine cotinine levels in smokers and nonsmokers**

**Weeks**	**Tocopherol (μg/ml plasma)**			
	**Nonsmokers**		**Smokers**	
	Placebo, n = 27	Palmvitee, n = 29	Placebo, n = 28	Palmvitee,n = 30
0	11.67 ± 3.19	11.75 ± 2.27	11.58 ± 3.19	11.39 ± 3.06
12	10.42 ± 2.17	14.22 ± 3.61*	10.27 ± 3.33	13.92 ± 4.17**
24	11.52 ± 2.94	14.56 ± 3.06*	11.53 ± 2.78	14.11 ± 3.89**
		**Tocotrienol (ng/ml)**		
0	62.49 ± 6.69	63.60 ± 5.67	60.05 ± 6.39	59.49 ± 4.21
12	63.50 ± 7.87	656.25 ± 142.50*	61.27 ± 3.62	570.22 ± 98.64**
24	60.65 ± 7.13	675.16 ± 150.00*	60.47 ± 3.00	587.68 ± 87.50**
		**CCR, (μg/mg creatinine)**		
	n = 16	n = 19	n = 21	n = 20
0	0.052 ± 0.017	0.046 ± 0.017	0.927 ± 0.734#	0.941 ± 0.700#
24	0.058 ± 0.030	0.047 ± 0.017	0.866 ± 0.754#	1.179 ± 0.738#
		**α**_**1**_**-antitripsin(g/L serum)**		
	n = 16	n = 19	n = 28	n = 30
0	1.48 ± 0.31	1.49 ± 0.34	1.99 ± 0.43#	1.94 ± 0.31#
24	1.47 ± 0.20	1.45 ± 0.24	1.69 ± 0.37	1.75 ± 0.39

Results are shown as mean ± SD. * indicates significant difference as compared to nonsmokers taking placebo (p < 0.01), ** indicates significant difference as compared to smokers taking placebo (p < 0.01), # indicates significant difference as compared to nonsmokers (p < 0.0001), CCR represents cotinine:creatinine ratio.

**Table 2:** Baseline levels of lymphocytes in nonsmokers and smokers according to the number of cigarettes per day

		**Nonsmokers n = 56**	**S1 (n = 7)**	**S2 (n = 34)**	**S3 (n = 15)**
Age	(y)	34 ± 8	35 ± 5	35 ± 6	36 ± 6
Body mass index	(kg/m^2^)	23.84 ± 3.99	24.11 ± 2.22	23.27 ± 3.26	23.55 ± 3.9822
Vitamin E	μg/ml	11.76 ± 3.17	11.66 ± 3.24	11.66 ± 3.24	11.66 ± 3.98
α_1_-antitrypsin	g/L serum	1.49 ± 0.32	1.98 ± 0.27*	1.96 ± 0.39*	1.94 ± 0.38*
CCR	μg/mg creatinine	0.049 ± 0.017	0.268 ± 0.244*	0.974 ± 0.759*	1.13 ± 0.56*
PHA	SI	70.54 ± 43.35	47.50 ± 34.63	89.05 ± 61.07	80.93 ± 55.85
Con A	SI	54.73 ± 38.35	33.33 ± 17.07	66.94 ± 41.32	57.13 ± 23.01
White cell count	(a.n. x 10^9^/L)	7.99 ± 1.74	9.83 ± 2.60*	8.71 ± 2.16	9.55 ± 1.96*
Lymphocytes	(a.n. x 10^9^/L)	3.25 ± 0.74	3.77 ± 1.45	3.50 ± 1.00	3.58 ± 1.01
	(%)	41.19 ± 7.76	37.68 ± 7.36	40.61 ± 6.74	39.83 ± 6.77
T cell	(a.n. x 10^9^/L)	2.05 ± 0.55	2.39 ± 0.10	2.36 ± 0.68	2.31 ± 0.71
	(%)	62.76 ± 7.54	63.14 ± 11.31	64.64 ± 7.32	64.67 ± 7.83
B cell	(a.n. x 10^9^/L)	0.38 ± 0.20	0.67 ± 0.51*	0.52 ± 0.29*	0.54 ± 0.17*
	(%)	11.13 ± 4.09	16.86 ± 6.20*	14.36 ± 4.20*	14.80 ± 4.28*
NK cells	(a.n. x 10^9^/L)	0.83 ± 0.35	0.67 ± 0.35	0.66 ± 0.31*	0.67 ± 0.33
	(%)	25.31 ± 903	19.00 ± 8.25*	19.34 ± 6.67*	18.20 ± 7.16*
CD4^+^	(a.n. x 10^9^/L)	0.92 ± 0.28	0.99 ± 0.30	1.18 ± 0.43*	1.14± 0.26*
	(%)	28.27 ± 6.43	27.29 ± 4.42	33.44 ± 5.46	31.47 ± 5.82
CD8^+^	(a.n. x 10^9^/L)	1.05 ± 0.39	1.17 ± 0.60	1.00 ± 0.34	1.049± 0.42
	(%)	31.53 ± 7.52	30.00 ± 7.81	28.89 ± 6.92	29.33 ± 7.16
CD4^+^/CD8^+^	(%)	0.97 ± 0.44	0.987 ± 0.383	1.22 ± 0.37*	1.16 ± 0.42

S1 refers to < 9 cigarettes/d, S2; 10-19 cigarettes/d and S3; >20 cigarettes/d. * indicates significant difference as compared to nonsmokers (p < 0.05).

### Lymphocyte proliferation

There was no difference in the lymphocyte proliferation after induction with mitogens PHA and Con A between the different groups (smoking vs nonsmoking) (Table 3). When the group of smokers divided into the number of cigarettes smoked per day, it seems to reduce in S1 group and increased in S2 and S3 group (Table 2). But it remained unaffected by palmvitee supplementation (Table 3). But there seemed to be a trend for the lymphocyte proliferation to increase at 24 weeks in the palmvitee-supplemented group induced with PHA (Table 3 &4).

**Table 3:** The effects of palmvitee supplementation on lymphocyte proliferative presented as stimulation index (S.I.) after induction with mitogen, PHA and Con A in nonsmokers and smokers

**Weeks**	**Con A (S.I)**			
	**Non-smokers**		**Smokers**	
	Placebo, n = 27	Palmvitee, n = 29	Placebo, n = 28	Palmvitee, n = 30
0	49.77 ± 36.42	59.17 ± 40.10	69.21 ± 41.11	52.72 ± 30.00
12	48.19 ± 28.62	51.03 ± 28.78	47.70 ± 29.73	59.50 ± 34.18
24	46.92 ± 23.64	55.10 ± 27.23	42.21 ± 26.59	42.93 ± 31.75
	**PHA (S.I)**			
0	66.72 ± 48.82	73.97 ± 38.35	91.96 ± 62.15	73.45 ± 53.44
12	70.59 ± 39.43	72.14 ± 28.88	65.74 ± 34.69	75.27 ± 35.85
24	69.15 ± 28.32	86.41 ± 41.53	69.39 ± 38.07	77.10 ± 59.44

Results are shown as mean ± SD.

**Table 4:** The effects of palmvitee supplementation on lymphocyte proliferative presented as stimulation index (S.I.) after induction with mitogen, PHA and Con A in nonsmokers and smokers according to the number of cigarettes per day

**Weeks**	**S1**		**S2**		**S3**	
	**Placebo, n = 4**	**Palmvitee, n = 3**	**Placebo, n = 17**	**Palmvitee,n = 19**	**Placebo, n = 7**	**Palmvitee,n = 8**
	**Con A (SI)**					
0	37.75 ± 15.73	24.50 ± 21.92	80.00 ± 48.12	55.26 ± 30.92	61.00 ± 15.41	53.75 ± 39.75
12	39.25 ± 15.09	55.00 ± 35.59	53.50 ± 35.02	59.32 ± 40.01	39.29 ± 20.77	61.63 ± 28.52*
24	36.00 ± 32.65	22.00 ± 24.56	46.24 ± 28.03	49.26 ± 35.55	36.00 ± 20.94	35.75 ± 26.99
	**PHA (SI)**					
0	58.00 ± 39.30	26.50 ± 6.36	73.17 ± 40.22	70.21 ± 44.99	67.29 ± 29.84	44.60 ± 28.53
12	60.50 ± 24.96	51.67 ± 30.62	69.44 ± 40.09	76.58 ± 35.20	60.29 ± 28.19	81.00 ± 38.53
24	59.25 ± 24.07	72.67 ± 69.82	74.71 ± 44.45	78.47 ± 68.50	62.29 ± 27.49	75.50 ± 32.96

Results are shown as means ± SD. * indicates significant difference as compared to nonsmokers.

### T-cell subsets and B cell percentage

Table 5 shows the baseline levels of white blood cells counts, lymphocytes and T-cell subsets in smokers and nonsmokers. Table 2 shows the baseline according the number of cigarettes smoked per day. When comparing immune parameters between smokers and nonsmokers, WBC counts was higher in smokers (n = 58) compared to nonsmokers (n = 56, p < 0.01) and unaffected by palmvitee supplementation (Table 6). No difference in the total number and percentage of lymphocytes and CD3^+^ cells was observed before and after palmvitee supplementation (Table 6). In separated group; S3, total CD3^+^ cells (T cell) increased significantly (p < 0.05) compared to nonsmokers before supplementation (Table 2) and remained unchanged in palmvitee-supplemented group (Table 7). The percentage of B cells in smokers was higher compared to nonsmokers (p < 0.0001) and also in S1 (p < 0.005), S2 (p < 0.0005) and S3 groups (p < 0.005) (Table 2). After palmvitee supplementation, B cells percentage was significantly increased in nonsmokers at 24 weeks (p < 0.05) but not in the smokers (Table 6). In S1 and S3 palmvitee-supplemented groups there was a trend of increment in B cells percentage. The increment was correlated at 12 (r = 0.98, 0.87) and 24 weeks (r = 0.98, 0.96) respectively.

**Table 5:** Baseline levels of lymphocytes in smokers and nonsmokers

		**Nonsmokers n = 56**	**Smokers n = 58**	
Age	(y)	34 ± 8	36 ± 6	NS
Body mass index (kg/m2)	(kg/m^2^)	23.84 ± 3.99	23.52 ± 3.42	NS
Vitamin E	μg/ml	11.76 ± 3.17	11.66 ± 3.24	NS
α_1_-antitrypsin	g/L serum	1.49 ± 0.32	1.96 ± 0.37	P < 0.0000001
CCR	μg/mg creatinine	0.049 ± 0.017	0.93 ± 0.31	P < 0.0000001
PHA	SI	70.54 ± 43.35	77.50 ± 55.34	NS
Con A	SI	54.73 ± 38.35	59.98 ± 36.29	NS
White cell count	(a.n. x 10^9^/L)	7.99 ± 1.74	9.06 ± 2.18	P < 0.01
Lymphocytes	(a.n. x 10^9^/L)	3.25 ± 0.74	3.58 ± 1.01	NS
	(%)	41.19 ± 7.76	39.83 ± 6.77	
.	NS			
T cell	(a.n. x 10^9^/L)	2.05 ± 0.55	2.31 ± 0.71	NS
	(%)	62.76 ± 7.54	64.67 ± 7.83	NS
B cell	(a.n. x 10^9^/L)	0.38 ± 0.20	0.54 ± 0.29	P < 0.00001
	(%)	11.13 ± 4.09	14.78 ± 4.48	P < 0.00001
NK cells	(a.n. x 10^9^/L)	0.83 ± 0.35	0.66 ± 0.31	P < 0.001
	(%)	25.31 ± 903	19.00 ± 6.88	P < 0.0001
CD4^+^	(a.n. x 10^9^/L)	0.92 ± 0.28	1.15± 0.38	P < 0.0001
	(%)	28.27 ± 6.43	32.19 ± 5.73	P < 0.0001
CD8^+^	(a.n. x 10^9^/L)	1.05 ± 0.39	1.04 ± 0.39	NS
	(%)	31.53 ± 7.52	29.14 ± 6.97	NS
CD4^+^/CD8^+^	(%)	0.97 ± 0.44	1.18 ± 0.38	P < 0.0001

a.n. represents actual number.

**Table 6:** **The effects of palmvitee supplementation on total white blood cells, lymphocytes, CD3**^**+**^**cells, B lymphocyte, in whole blood of nonsmokers and smokers**

**Weeks**	**WBC**	**Lymphocytes**		**CD3+ cells**		**B lymphocyte**	
	**a.n. x 10**^**9**^**/L**	**%**	**a.n. x 10**^**9**^**/L**	**%**	**a.n. x 10**^**9**^**/L**	**%**	**a.n. x 10**^**9**^**/L**
	**Non-smokers (placebo, n = 27)**						
0	7.62 ± 1.89	41.96 ± 9.34	3.12 ± 0.77	63.54 ± 7.03	1.99 ± 0.60	10.19 ± 4.06	0.35 ± 0.26
12	7.29 ± 2.10	42.39 ± 6.12	3.04 ± 0.84	65.96 ± 6.70	2.00 ± 0.56	10.89 ± 3.56	0.33 ± 0.14
24	7.14 ± 1.53	41.85 ± 8.85	2.94 ± 0.72	64.27 ± 7.12	1.88 ± 0.50	11.00 ± 3.88	0.32 ± 0.12
	**Non-smokers (palmvitee, n = 29)**						
0	8.32 ± 1.55	40.52 ± 6.17	3.36 ± 0.70	62.07 ± 8.02	2.09 ± 0.52	11.97 ± 4.00	0.40 ± 0.13+
12	8.27 ± 2.08	40.39 ± 6.09	3.33 ± 0.74	62.72 ± 7.60	2.10 ± 0.55	12.45 ± 4.67	0.41 ± 0.16+
24	8.02 ± 1.50	38.05 ± 5.80+	3.05 ± 0.77	61.66 ± 14.23	1.96 ± 0.55	13.72 ± 4.53+	0.41 ± 0.15+
	**Smokers (placebo, n = 28)**						
0	9.74 ± 2.31	40.44 ± 7.39	3.91 ± 1.14	63.50 ± 7.87	2.47 ± 0.71	15.54 ± 5.06	0.64 ± 0.38
12	9.38 ± 2.45	40.53 ± 8.20	3.76 ± 1.16	63.82 ± 8.34	2.40 ± 0.65	16.14 ± 4.70	0.59 ± 0.33
24	8.81 ± 2.30	38.00 ± 8.68	3.30 ± 1.04	66.11 ± 10.78	2.14 ± 0.63	15.25 ± 4.40	0.52 ± 0.29
	**Smokers (palmvitee, n = 30)**						
0	8.43 ± 1.86	39.27 ± 6.20	3.27 ± 0.75	65.77 ± 7.75	2.16 ± 0.69	14.06 ± 3.81	0.45 ± 0.15
12	8.13 ± 2.29	40.07 ± 7.04	3.19 ± 0.72	66.27 ± 6.59	2.13 ± 0.58	14.30 ± 4.09	0.45 ± 0.16
24	8.06 ± 2.10	37.68 ± 7.29	3.01 ± 0.88	66.83 ± 7.28	2.02 ± 0.69	14.40 ± 4.03	0.43 ± 0.16

Results are shown as mean ± SD. + indicates significant difference as compared to non-smokers taking placebo (p < 0.05), a.n. represents actual number.

**Table 7:** **The effects of palmvitee supplementation on total white blood cells, lymphocytes, CD3**^**+**^**cells, B lymphocyte, in whole blood of nonsmokers and smokers according to the number of cigarettes per day**

**Weeks**	**WBC**	**Lymphocytes**		**CD3**^**+**^**cells**		**B lymphocyte**	
	**a.n. x 10**^**9**^**/L**	**%**	**a.n. x 10**^**9**^**/L**	**%**	**a.n. x 10**^**9**^**/L**	**%**	**a.n. x 10**^**9**^**/L**
	**S1; 1-9 cigarettes/d (placebo, n = 4)**						
0	9.80 ± 2.07	37.25 ± 8.59	3.79 ± 1.65	59.75 ± 13.25	2.21 ± 0.81	17.75 ± 8.42	0.76 ± 0.70
12	10.05 ± 3.10	38.63 ± 10.59	3.94 ± 2.08	58.00 ± 11.58	2.36 ± 0.93	18.00 ± 6.38	0.81 ± 0.73
24	9.43 ± 2.67	36.05 ± 10.39	3.56 ± 1.92	54.75 ± 21.93	1.91 ± 0.96	15.25 ± 6.90	0.63 ± 0.52
	**S1; 1-9 cigarettes/d (palmvitee, n = 3)**						
0	9.87 ± 3.72	38.27 ± 7.15	3.75 ± 1.52	67.67 ± 8.14	2.62 ± 1.40	15.67 ± 2.31	0.56 ± 0.13
12	6.73 ± 1.10	39.57 ± 5.75	2.64 ± 0.35	69.00 ± 6.08	1.80 ± 0.11	17.67 ± 4.04	0.48 ± 0.16
24	6.53 ± 0.76	35.20 ± 11.15	2.29 ± 0.70	65.33 ± 5.77	1.47 ± 0.35	19.00 ± 2.65	0.45 ± 0.18
	**S2; 10-19 cigarettes/d (placebo, n = 17)**						
0	9.31 ± 2.53	42.85 ± 6.63	3.96 ± 1.20	64.24 ± 6.28	2.54 ± 0.76	14.82 ± 4.25	0.63 ± 0.37
12	8.92 ± 2.61	41.19 ± 7.34	3.62 ± 0.99	65.71 ± 7.08	2.38 ± 0.62	15.47 ± 3.68	0.52 ± 0.28
24	8.22 ± 2.38	40.26 ± 8.70	3.23 ± 0.95	68.12± 7.03	2.18 ± 0.63	14.82 ± 3.59	0.50 ± 0.2
	**S2; 10-19 cigarettes/d (palmvitee, n = 19)**						
0	8.17 ± 1.65	38.59 ± 6.35	3.10 ± 0.54	65.00 ± 8.30	2.00 ± 0.51	13.95 ± 4.24	0.42 ± 0.14
12	8.11 ± 2.36	38.51 ± 7.34	3.02 ± 0.60	65.74 ± 7.34	2.01 ± 0.52	13.63 ± 3.52	0.41 ± 0.14
24	7.64 ± 2.02	37.22 ± 8.70	2.81 ± 0.74	67.26 ± 7.01	1.89 ± 0.55	13.37 ± 3.99	0.38 ± 0.15
	**S3; >20 cigarettes/d (placebo, n = 7)**						
0	10.76 ± 1.75	36.42 ± 7.01	3.87 ± 0.81	63.86 ± 8.65	2.46 ± 0.58	16.00 ± 5.16	0.60 ± 0.17
12	10.11 ± 1.58	39.99 ± 9.93	3.99 ± 1.10	62.57 ± 8.83	2.49 ± 0.68	16.71 ± 6.26	0.64 ± 0.23
24	9.90 ± 1.58	33.66 ± 6.62	3.31 ± 0.77	67.71± 7.09	2.18 ± 0.44	16.29 ± 5.22	0.50 ± 0.18
	**S3; >20 cigarettes/d (palmvitee, n = 8)**						
0	8.50 ± 1.54	41.23 ± 6.12	3.50 ± 0.70	66.88 ± 7.95	2.37 ± 0.51	13.75 ± 3.97	0.48 ± 0.13
12	8.70 ± 2.06	43.96 ± 6.03	3.79 ± 0.74	66.50 ± 7.53	2.55 ± 0.55	14.63 ± 4.63	0.55 ± 0.16
24	9.64 ± 1.49	39.71 ± 5.75	3.75 ± 0.76	66.38 ± 14.10	2.54 ± 0.54	15.13 ± 4.49	0.56 ± 0.15

Results are shown as mean ± SD.

The percentage of NK cells were lower in smokers compared to nonsmokers (p < 0.0001) at baseline (S1: p < 0.05, S2: p < 0.005, S3: p < 0.01) and after palmvitee supplementation (Table 8 and Table 9). CD4^+^ in nonsmokers were lower than smokers at the beginning of the study (p < 0.0001). Total CD4^+^ in smokers were increased according to number of cigarettes per day. CD8^+^ percentage did not show any significant difference among the groups. CD4^+^/CD8^+^ ratio was found to be significantly increased (p < 0.001) in smokers compared to nonsmokers at baseline (Table 5) and also in S2. However, no significant changes were observed after palmvitee supplementation for both smokers and nonsmokers (Table 9). There were also no changes observed in these parameters at the different times of supplementation.

**Table 8:** **The effects of vitamin E supplementation on NK cells, CD4**^**+**^**cells, CD8**^**+**^**cells and CD4**^**+**^**/CD8**^**+**^**in whole blood of nonsmokers and smokers**

**Weeks**	**NK cells**		**CD4**^**+**^		**CD8**^**+**^		**CD4**^**+**^**/CD8**^**+**^
	**%**	**a.n. x 10**^**9**^	**%**	**a.n. x 10**^**9**^	**%**	**a.n. x 10**^**9**^	**%**
	**Non-smokers (placebo, n = 27)**						
0	25.00 ± 9.44	0.73 ± 0.34	29.07 ± 6.16	0.91 ± 0.28	31.69 ± 7.41	0.99 ± 0.41	0.99 ± 0.40
12	22.85 ± 7.74	0.70 ± 0.35	30.81 ± 6.75	0.94 ± 0.35	31.19 ± 8.03	1.00 ± 0.36	1.02 ± 0.46
24	24.72 ± 7.94	0.75 ± 0.32	31.27 ± 6.87	0.92 ± 0.32	32.07 ± 8.30	0.94 ± 0.36	1.07 ± 0.48
	**Non-smokers (palmvitee, n = 29)**						
0	25.59 ± 8.80	0.86 ± 0.36	27.55 ± 6.68	0.92 ± 0.29	31.38 ± 7.75	1.07 ± 0.30	0.96 ± 0.48
12	24.59 ± 8.45	0.81 ± 0.33	27.62 ± 7.72	0.92 ± 0.33	30.28 ± 6.75	1.03 ± 0.35	0.97 ± 0.47
24	24.72 ± 8.78	0.72 ± 0.42	30.17 ± 9.35	0.90 ± 0.30	31.00 ± 6.72	0.96 ± 0.37	1.03 ± 0.53
	**Smokers (placebo, n = 28)**						
0	19.54 ± 7.50+	0.76 ± 0.39	32.18 ± 5.98+	1.24 ± 0.44+	27.82 ± 5.36+	1.08 ± 0.34	1.22 ± 0.38+
12	19.46 ± 8.55+	0.75 ± 0.45	33.07 ± 7.32+	1.23 ± 0.39+	27.46 ± 6.01+	1.05 ± 0.36	1.24 ± 0.40+
24	19.18 ± 10.93+	0.64 ± 0.46	35.14 ± 7.93+	1.14 ± 0.44+	28.04 ± 7.34	0.93 ± 0.27	1.25 ± 0.36+
	**Smokers (palmvitee, n = 30)**						
0	18.48 ± 6.32++	0.58 ± 0.17++	32.20 ± 5.54++	1.03 ± 0.29	30.37 ± 8.09	1.00 ± 0.45	1.13 ± 0.39
12	17.87 ± 6.50++	0.55 ± 0.21++	33.20 ± 5.56++	1.07 ± 0.29++	30.17 ± 8.21	0.98 ± 0.42	1.21 ± 0.44++
24	19.03 ± 7.82	0.57 ± 0.27	33.90 ± 5.56++	1.03 ± 0.37	30.67 ± 7.61	0.92 ± 0.38	1.19 ± 0.43++

Results are shown as mean ± SD. a.n. represents actual number. + indicates significant difference as compared to nonsmokers taking placebo (p < 0.05). ++ indicates significant difference as compared to nonsmokers taking palmvitee (p < 0.05).

**Table 9:** **The effects of vitamin E supplementation on NK cells, CD4**^**+**^**cells, CD8**^**+**^**cells and CD4**^**+**^**/CD8**^**+**^**in whole blood of nonsmokers and smokers according to the number of cigarettes per day**

**Weeks**	**NK cells**	**CD4**^**+**^		**CD8**^**+**^		**CD4**^**+**^**/CD8**^**+**^	
	**%**	**a.n. x 10**^**9**^	**%**	**a.n. x 10**^**9**^	**%**	**a.n. x 10**^**9**^	**%**
	**S1; 1-9 cigarettes/d (placebo, n = 4)**						
0	21.50 ± 9.57	0.78 ± 0.45	26.25 ± 5.44	0.93 ± 0.22	28.25 ± 9.11	1.09 ± 0.51	1.05 ± 0.50
12	22.00 ± 12.73	0.87 ± 0.65	25.25 ± 3.20	0.96 ± 0.41	27.25 ± 9.25	1.07 ± 0.55	1.02 ± 0.43
24	31.75 ± 22.78	1.09 ± 0.97	23.75 ± 7.27	0.83 ± 0.40	25.50 ± 11.85	0.87 ± 0.42	0.99 ± 0.22
	**S1; 1-9 cigarettes/d (palmvitee, n = 3)**						
0	15.67 ± 6.11	0.53 ± 0.04	28.67 ± 3.06	1.07 ± 0.43	32.33 ± 6.66	1.28 ± 0.79	0.90 ± 0.20
12	13.67 ± 1.53	0.37 ± 0.09	34.67 ± 2.08	0.92 ± 0.16	30.67 ± 4.73	0.79 ± 0.46	1.16 ± 0.23
24	18.33 ± 6.43	0.45 ± 0.25	29.67 ± 3.79	0.69 ± 0.26	31.00 ± 6.24	0.68 ± 0.10	0.99 ± 0.29
	**S2; 10-19 cigarettes/d (placebo, n = 17)**						
0	19.59 ± 6.75	0.76 ± 0.38	34.18 ± 6.15	1.37 ± 0.52	27.76 ± 4.83	1.07 ± 0.32	1.30 ± 0.39
12	20.12 ± 8.37	0.71 ± 0.39	35.65 ± 7.55	1.29 ± 0.41	27.24 ± 5.84	1.01 ± 0.29	1.33 ± 0.40
24	17.47 ± 6.69	0.55 ± 0.25	37.06 ± 7.04	1.21 ± 0.49	28.94 ± 7.26	0.93 ± 0.25	1.31 ± 0.38
	**S2; 10-19 cigarettes/d (palmvitee, n = 19)**						
0	19.11 ± 6.78	0.57 ± 0.20	32.79 ± 4.85	1.02 ± 0.26	29.89 ± 8.38	0.93 ± 0.35	1.15 ± 0.34
12	18.42 ± 7.27	0.53 ± 0.20	33.05 ±5.78	1.01 ± 0.26	30.05 ± 7.71	0.93 ± 0.33	1.20 ± 0.45
24	18.58 ± 7.88	0.52 ± 0.26	34.95 ± 5.02	0.99 ± 0.34	30.74 ± 7.26	0.85 ± 0.28	1.23 ± 0.47
	**S3; >20 cigarettes/d (placebo, n = 7)**						
0	18.29 ± 9.01	0.74 ± 0.47	30.71 ± 2.81	1.18 ± 0.21	27.71 ± 5.06	1.08 ± 0.35	1.15 ± 0.30
12	18.43 ± 7.46	0.76 ± 0.52	31.29 ± 4.57	1.21 ± 0.27	28.14 ± 5.30	1.13 ± 0.42	1.14 ± 0.38
24	16.14 ± 5.84	0.61 ± 0.39	37.00 ± 4.86	1.15 ± 0.25	27.29 ± 4.96	1.01 ± 0.45	1.24 ± 0.37
	**S3; >20 cigarettes/d (palmvitee, n = 8)**						
0	18.13 ± 8.72	0.60 ± 0.36	32.13 ± 6.63	1.09 ± 0.29	30.75 ± 7.68	1.09 ± 0.37	1.17 ± 0.48
12	18.13 ± 8.37	0.67 ± 0.32	33.00 ± 7.65	1.24 ± 0.33	30.25 ± 6.69	1.17 ± 0.35	1.25 ± 0.46
24	20.38 ± 8.70	0.73 ± 0.42	33.00 ± 9.27	1.24 ± 0.29	30.38 ± 6.66	1.17 ± 0.37	1.19 ± 0.15

Results are shown as mean ± SD.

A significant positive correlation was observed in smokers when comparing total white blood cells count and total B cells count at 0 week (r = 0.57, p < 0.05) (Table 2). Also a significant positive correlation was found between TWBC count and total CD4^+^ cell count before starting supplementation (r = 0.59, p < 0.05).

## Discussion

Avoidance of tobacco and smoking cessation represent the best method overcoming disease cases such as cancer, chronic obstructive pulmonary disease (COPD) and chronic heart disease (CHD). These approaches may not be successful for some smokers. It might be due to their attitude or self discipline to stop smoking. Supplementation of vitamins or minerals can be used as an alternative to increase the antioxidant levels and immune system in smokers. In this study, report the effect of a randomized single-blind placebo-controlled trial on the immune response of cigarette smokers after palmvitee supplementation. Supplementation of 200 mg/d palmvitee for 24 weeks to the smokers and nonsmokers increase total plasma vitamin E levels in plasma indicating compliance of the study subjects. When this total vitamin E was separated according to its type –tocopherol and tocotrienol, both were also increased with supplementation. The concentration of tocopherol was higher in plasma as compared to tocotrienol because of the action of hepatic α-tocopherol transfer protein (α-TTP). α-TTP selectively chooses α-tocopherol for enrichment of nascent very low density lipoproteins (VLDL) [25]. During VLDL catabolism in the circulation, α-tocopherol is transferred to all the other plasma lipoproteins. Although, the dose used in the study was 200 mg/d vitamin E, only 40% of palmvitee is α-tocopherol while 60% consisted of tocotrienols. Whereas tocotrienols are transported and distributed differently in different tissues according to their roles in cellular function [26]. For example, skin contained 15% tocotrienols and only 1% γ-tocopherol. Although, the accumulation of tocotrienols may be low, but they may exert substantial antioxidant effects [27]. Studied by Maniam *et al.*[28] in rats showed that tocotrienol gives better protective effect against free radical damage in the femur compared to alpha-tocopherol. Reported by Saito *et al.*[29] the uptake of alpha-tocotrienol in Jurkat cells was found to be 2.2 fold higher than alpha-tocopherol after incubation for 72 hours. Also, it was found that the initial rate of cellular uptake of alpha-tocotrienol was 70-fold higher than alpha-tocopherol [30].

There was no significant difference in plasma vitamin E levels for tocopherol and tocotrienol between smokers and nonsmokers was observed. Previous study reported by Wallstrom *et al.*[31] demonstrated that serum vitamin E levels (α-tocopherol) were similar in smokers vs. nonsmokers and only associated with dietary supplements, not with foods. Whereas when the smokers were grouped according to number of cigarettes smoked per day, it gave different result. Antioxidant vitamin intakes were significantly higher in nonsmokers than in light (1-20 cigarettes/day) and heavy smokers (>20 cigarettes/day) [32]. Exposure of human plasma *in vivo* to the gas phase of cigarette smoke will cause degradation of vitamin E [33]. Fractional disappearance rate of α-tocopherol in smokers were faster, and its half-lives were shorter than in nonsmokers [34]. This may lead to insufficient levels of vitamin E and was suggested to have an effect on the immune status of these individuals. Meydani *et al.*[35] showed via a placebo-controlled, double-blind study using healthy elderly individuals for 235 days that after varying the dose of dl-α-tocopherol supplementation, it was found that a dose of 200 mg/d caused the highest percent increase in delayed type hypersensitivity, suggesting that 200 mg/d might be a threshold level for the immunostimulatory effect of vitamin E. It also supported by Lee and Man-Fan [19] in supplementing healthy ethnic Chinese men and women with *dl*-α-tocopherol E (233 mg/d) for 28 days.

Comparison of lymphocyte proliferation measured as stimulation index induction with mitogen PHA and Con A did not show any differences between smokers and nonsmokers were also unaffected by supplementation of palmvitee (Tables 5 and 3). But lymphocyte response to the mitogen PHA also seemed to be increased with palmvitee supplementation. This observation differed from that reported by Meydani *et al.*[18] where only Con A stimulated mitogenic response increased in the vitamin E supplemented group. Reported by Lee and Man-Fan Wan [19] supplementation of vitamin E in healthy individual subjects increased lymphocyte proliferation both in the presence and absence of mitogen challenge and also the increasing of immunological subsets. But there was a confounding factor (gender) because hormonal changes play a role in the regulation of the immune response [36] Studied by Radhakrishnan *et al.*[37] in healthy human volunteers supplemented with 200 mg of tocotrienol-rich fraction or alpha-tocopherol showed no changes observed in the production of IL-4 or interferon-γ by Con A-stimulated lymphocytes. In this study, palmvitee supplementation also did not affect T-cell subsets and a similar finding was reported by Meydani *et al.*[35]. Cigarette smoke is reported to contain many oxidising species [38] and smokers incur a high and sustained free radical load. Smokers may need a higher dose of vitamin E than 200 mg/day to overcome the free radical load and increase the immune system. A study using monkeys have shown that a low dose cigarette smoke (human equivalent of 1 pack day) affect the response of spleens cells to either PHA or LPS whereas a heavy dose (human equivalent of 3 pack day) for 4-8 years caused a significant reduction in their natural NK-mediated lytic activity and a decreased response to Con A [39]. It is possible that the subjects were not heavy smokers to cause any changes to the lymphocytes proliferation activity. Studied by Thatcher *et al.*[40] showed that at the higher dose of mainstream cigarette smoke (MSC) exposure (600 mg/m3 total suspended particulates (TSP) suppresses the antigen-specific proliferation and cytokine production by T-cell than low dose of MSC (77 mg/m3 TSP).

Difference between smokers and nonsmokers in baseline volumes of certain immune parameters measured were also observed these include higher total white cell counts, CD4^+^ cells and CD4^+^/CD8^+^ in smokers and lower number of natural killer cells. These differences in T-cells populations have also been reported in different studies in different countries. For example, the results obtained by Tollerud *et al.*[41] have shown that CD4^+^ T-cells but not in CD8^+^ T-cells, CD3^+^ T-cells or CD19^+^ B-cells were higher in smokers compared to nonsmokers. Other T-cells subsets such as memory and naïve T-cells subpopulations were also increased in smokers [42]. The reduced NK cell observed in smokers is in agreement with another study by Moszczynski *et al.*[43]. The reduction of NK cells in smokers correlated with a reduction in immune surveillance against tumors and viral infections [44] and maybe a contributing factor to development of malignancy. Studied by Lu *et al.*[45] in mice showed the consistent finding where that cigarette smoke suppressed NK activation and lead cell transformation and cancer formation. But other several studies reported that there were no changes in NK cells [46,47] and no significant differences in NK cell percentage but NK tumoricidal activity was significantly higher. Tanigawa *et al.*[42] reported an increase in CD4^+^ cells in smokers compared with nonsmokers which was in agreement with the results obtained in this study. They also reported that the increase in the number of CD4 + CD29+ (helper inducer) T lymphocytes is responsible for the increase in total CD4^+^ T lymphocytes in smokers. This may be due to continuous local inflammation in the respiratory system induced by chronic smoking. Another possible explanation is that antigenic substances present such as glycoproteins present in some cigarettes may act as an antigen leading to an increase in CD4 + CD29+ T lymphocytes as the tobacco glycoprotein induces the production of interleukin (IL) 1 alpha and IL-1 beta by peripheral blood and adherent cells. Tobacco glycoprotein is a potent immunostimulatory compound that has been isolated from cigarette smoke [48] where it has been shown to be antigenic in humans.

The B cells percentage was higher in smokers than nonsmokers and this was also reported by Mili *et al.*[49] which was attributed to an increase in CD4^+^ in smokers. It is possible that in this study, CD4^+^ was induced by tobacco glycoprotein and provided a signal to B cells to produce antibodies. Palmvitee supplementation was found to increase B cells in nonsmokers only. This observation could be due to the immunoenhancing effect of vitamin E which acts by a reducing prostaglandins synthesis and or decreasing free radical formation [50]. This finding however differed with that of Meydani *et al.*[35] who reported that vitamin E supplementation had no effect on immunoglobulin levels or levels of T and B cells in healthy elderly subjects. The difference observed could be due to the different age groups of the subjects and it is well established that the immune response is influenced by age. The increment of B cells and CD4^+^ cell correlated with the higher TWBC in smokers.

A similar pattern was observed when the immune parameters were measured in terms of packed years of exposure rather than cigarettes per day. Tanigawa *et al.*[42] also reported higher CD4^+^ lymphocytes in smokers but no differences in CD8^+^, CD19^+^ B lymphocytes and CD16^+^ NK cells. The findings rather of the two parameters were however contrary to our present findings where B lymphocyte was raised while NK cells were decreased. However, Tanigawa *et al.*[42] reported the results of only 8 male smokers whereas in this study other were 58 smokers which were also age-matched with the nonsmokers. Another studied by Moszczynski *et al.*[43] who grouped the subjects in terms of less and over 10 years of smoking gave different pattern of result. CD4^+^, CD8^+^ and NK cells increased in smoked-group less than 10 years whereas reduced in smoked-group over 10 years. No changes were observed in B lymphocytes. The highly significant (p < 0.001) reduction in NK cells could also explain increased risk to cancer formation due to a decrease in cellular mediated immune protection/surveillance.

It was interesting to note that while supplementation had no effect on the lymphocyte proliferation as well as on total white cells, total lymphocytes, and total T-cells in both smokers and nonsmokers, palmvitee supplementation was observed to cause an increase (p < 0.05) in the number of B-cells in nonsmokers. This was --- betted to the response beneficial effect of palmvitee in enhancing immune response. However, in smokers B-cells numbers were unaffected by supplementation possibly because of the already raised basal values caused by smoking. This is the only immune parameter which was affected by palmvitee supplementation. Other parameter remained low with supplementation, whilst the high CD4^+^ and lower CD8^+^ percentage of cells remained unchanged with supplementation. This lack of effect could be due to the dosage used which was only 200 mg/d compared to 800 mg/d as reported by Meydani *et al.*[35] in his study in the elderly population.

Urinary cotinine/creatinine standardized as μg/mg, a stable metabolite of nicotine, were increased significantly in smokers indicating the active smoking status of subjects compared to nonsmokers [51]. Obviously when smoking group divided into the number of cigarette smoked per day urinary cotinine increase accordingly to the number of cigarette smoked per day. Also serum α_1_-antitrypsin was significantly higher in smokers, again indicating the smoking status of these subjects. Elevations in α_1_-antitrypsin were significantly associated with the impairment of pulmonary function to smoking. Supplementation with palmvitee demonstrated no changes for both CCR and α_1_-antitrypsin concentration in smokers as compared to the placebo group. It showed that might be there were no interaction between CCR and α_1_-antitrypsin with palmvitee that can reduced their level in the blood.

## Conclusions

In conclusions, cigarette smoking is associated with an increase in CD4^+^ cell, B cells and a decrease in CD8^+^ and NK cells. An increase in CD4^+^ cell and B cells correlated with higher TWBC counts in smokers. Although vitamin E increased in supplemented smokers, there were no changes in T-cell profile except for an increase in the number of B cells in nonsmokers. In smokers, proliferation of lymphocytes after stimulation by PHA tended to be increased with time of vitamin E supplemented but not after exposure to mitogen Con A. Vitamin E might not reach the optimal levels to modulate or improve the immune status.

## Abbreviations

HPLC: High performance liquid chromatography; LTT: Lymphocyte transformation test; PHA: Phytohemagglutinin; LPS: Lipopolysacarides; Con A: Concanavalin A; TWBC: Total white blood cells; PGE_2_: Prostaglandin E2; BHT: Butylated hydroxytoluene; UV: Ultraviolet; CCR: Cotinine to creatinine; PBMN: Peripheral blood mononuclear cells; CPM: Counts per min; PBS: Phosphate buffer solution; SD: Standard deviation; Vs: Versus; NK: Natural killer; COPD: Chronic obstructive disease; CHD: Chronic heart disease; α-TTP: Hepatic α-tocopherol transfer protein; VLDL: Very low density lipoprotein; MSC: Mainstream cigarette smoke; IL: Interleukin; CD: Cluster of differentiation.

## Competing interests

Dr Abdul Gapor Md Top is an employee of the Malaysian Palm Oil Board as the other authors declare no competing interests.

## Authors’ contributions

The contribution of each author was as follows: ZJ undertook the overall management of the study and most of the laboratory and statistical analysis and drafting of the manuscript. AAL participate in giving the briefing about the study to the volunteers and taking the volunteers’ blood. AGMT involved in supplying the palm oil vitamin E (palmvitee) for the research. WZWN contributed to the design and subsequent finalization of the manuscript. All authors read and approved the final manuscript.
